# Evaluation of 3D planning in orthognathic surgery

**DOI:** 10.2340/aos.v84.44805

**Published:** 2025-10-10

**Authors:** Klas Strindlund, Payam Farzad, Lars Rasmusson, Carina Cardemil

**Affiliations:** a Department of Oral and Maxillofacial Surgery and Jaw Orthopedics, Karolinska University Hospital, Stockholm, Sweden; b Maxillofacial Unit, Linkoping University Hospital, Linkoping, Sweden; c Department of Oral and Maxillofacial Surgery, Sahlgrenska Academy at University of Gothenburg, Gothenburg, Sweden; d Department of Biomaterials, Institute of Clinical Sciences, Sahlgrenska Academy at University of Gothenburg, Gothenburg, Sweden

**Keywords:** virtual surgical planning, orthognathic surgery, 3D planning

## Abstract

**Objective:**

The use of virtual surgical planning (VSP) in orthognathic surgery is increasing. The aim of this study was to investigate which type of dentofacial deformity makes the surgeon choose VSP and if VSP influences the choice of surgical techniques. Furthermore, we sought to evaluate if use of VSP affects surgical time and blood loss during surgery.

**Material and methods:**

Patients having undergone orthognathic surgery and registered in the Swedish quality registry for orthognathic surgery (NROK) between 2018 and 2019 were eligible for inclusion in this study. An evaluation of the usage of VSP was performed together with an analysis of operation time and bleeding during surgery. A total of 862 patients were evaluated.

**Results:**

Out of the 862 patients who had orthognathic surgery, 224 were VSP cases. Patients diagnosed with maxillary retrusion (p = 0.0024), maxillary vertical hyperplasia (p < 0.001), mandibular protrusion (p = 0.0031), laterogenia (p < 0.001) or craniofacial deformity (p = 0.0033) were more often digitally planned. Patients who underwent VSP and bimaxillary surgery showed a tendency of reduced operating time andless bleeding during surgery; however, the results were inconclusive.

**Conclusion:**

The present study shows that VSP was used more frequently in patients with maxillary retrusion, maxillary vertical hyperplasia, mandibular protrusion, laterogenia, and craniofacial deformity in comparison with other diagnoses. Alongside the findings that VSP cases had more diagnoses per patient and that a larger number of surgical procedures were performed in this group, this indicates a preference for using digital planning in more complex orthognathic cases.

## Background

**Figure 1:**
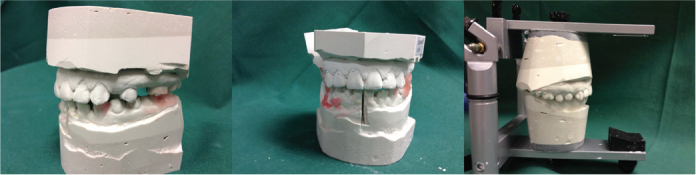
Traditional surgical planning (TSP)

**Figure 2:**
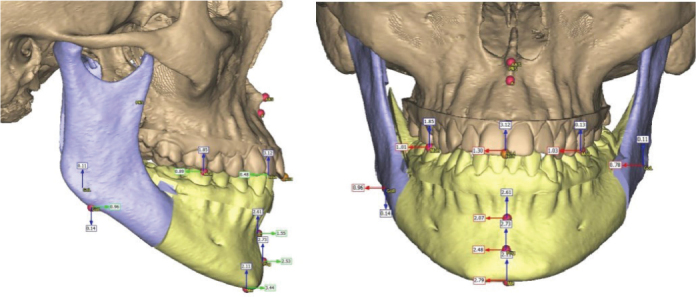
Virtual Surgical Planning (VSP).

Orthognathic surgery is performed to correct dentofacial deformities and to enhance patient occlusion, airway, and facial esthetics. This procedure has been shown to improve quality of life and self-esteem among patients [1]. To optimize treatment outcomes, accurate preoperative planning is of utmost importance. Therefore, new methods for planning orthognathic surgery are continuously being developed [2, 3]. Traditional surgical planning (TSP) involves evaluation of two-dimensional (2D) cephalograms, dental casts, and the use of manual mock surgeries with wax bites and model surgery (Figure 1). This method, which is more dependent on operator experience and manual accuracy, has shortcomings in terms of visualizing complex three-dimensional relationships. Nevertheless, it has been the ‘gold standard’ in orthognathic surgical planning for over 100 years. However, in the past decade it has been increasingly challenged by alternative novel approaches [3–5]. During the 1970s, the Computed Tomography (CT) technique was invented by Sir Godfrey Hounsfield followed by the 3D printers, which started to emerge during the 1980s, together with Computer Aided Design/Computer Aided Manufacturing (CAD/CAM) [4]. These technological advancements cleared the way for what today is referred to as Virtual Surgical Planning (VSP) [5, 6] (Figure 2). This involves the use of 3D imaging, virtual simulation software, and sometimes intraoral scanning and 3D-printed surgical guides, allowing for detailed planning and visualization of the desired skeletal movements before surgery. The virtual environment enables precise segmentation, alignment, and simulation of osteotomies and repositioning of skeletal segments.

Within the field of Oral and Maxillofacial surgery, VSP is used routinely in orthognathic surgical planning, pre-surgical planning in facial trauma surgery and also in reconstructive surgery of the facial skeleton [7].

VSP in orthognathic surgery has seen a steady increase in usage. Although a learning curve has been identified, studies have shown that VSP improves surgical accuracy, reduces planning time for clinicians, and can subsequently lead to shorter operation times [7–10]. Possibilities are emerging with the usage of VSP that were not possible just a few years ago, such as the application of artificial intelligence in surgical planning [11]. It is also known that digital planning shortens the time used for planning of the surgery and treatment in maxillofacial reconstruction [12, 13]. However, the implications of VSP on orthognathic surgery, particularly regarding clinical outcomes such as operative time and intraoperative bleeding, remain insufficiently investigated [14]. It has been shown that VSP leads to shorter surgical time when used in conjunction with pre-bent plates [15]. However, there are only a few publications that have analyzed factors influencing treatment time without pre-bent plates. Narita et al. investigated a total of 45 patients, comparing cases planned with or without 3D printed models and could show that the group where a 3D model was available had significantly shorter operative time and a non-significant reduction in bleeding during surgery [16]. Another systematic review showed a tendency towards shorter surgeries in VSP cases [14].

The main objective of this study was to investigate which diagnoses influenced the surgeons’ decision to choose VSP. Secondary objectives were to investigate how surgical choices and techniques were affected by VSP and also if VSP shortened the surgical time or reduced blood loss during surgery. Data were collected from the Swedish quality registry for orthognathic surgery (NROK).

## Materials and methods

This is a retrospective cohort study with data from the Swedish quality registry NROK, collected between 2018 and 2019. NROK was launched nationwide in January 2018 [17]. It consists of a 5-minute online registration form completed by the clinician immediately after surgery, and another shorter form to be completed at 1-year follow-up. After surgery, the surgeon provides information about the patient’s health condition, smoking habits, dentofacial diagnosis, orthodontic treatment, operation type, type of fixation, operation time, bleeding during surgery, and perioperative medication. At the 1-year follow-up, the clinician provides information regarding sensory disturbances, postoperative infection, re-operation, and patient satisfaction.

The clinician also registers if VSP was utilized; however, there is no information provided on the type of VSP or how the final occlusion was set, either virtually or scanned from the dental casts. The NROK registry is based on the national population of Sweden and has been reported to have a high coverage rate [17].

### Data collection

All patients who underwent surgery during the study period were included. A total of 862 patients were evaluated from the NROK registry from the years 2018–2019. When evaluating which diagnoses lead to the usage of VSP, we analyzed all 862 patients without any missing values. Out of these patients, 224 had been virtually planned, and these were further analyzed based on the diagnosis received before treatment. From the same set of data, calculations were also made about which treatment options were used more often in combination with VSP.

A separate analysis was also made to evaluate the surgical outcomes, where only individuals who underwent Le Fort I (LFI) surgery in combination with Bilateral Sagittal Split Osteotomy (BSSO), without any concomitant procedure, were further investigated. This group consisted of a total of 275 individuals, where 126 were virtually planned and 149 were planned with TSP.

Bleeding during surgery was recorded by the operating clinician directly after the procedure and documented in milliliters (mL). No standard protocol for measurement method (e.g. suction volume minus irrigation) was enforced across centers. Facial anomalies were classified by the operating clinician according to the NROK (Swedish National Registry for Orthognathic Surgery) diagnostic categories. Only patients with complete data for both surgical planning method (VSP or TSP) and outcome variables (bleeding, operative time) were included in the final analyses.

### Statistical analyses

Statistical analyses were performed using SPSS Version 28.0.1.0 (142). P-values below 0.05 were considered statistically significant. To calculate the p-values and standard deviations when comparing operative time and bleeding between VSP and TSP cases, the Fisher permutation test was used.

The study was approved by the Swedish Ethical Review Authority, Dnr: 2021-01083.

## Results

The 862 patients included in this study had a total of 1,438 diagnoses of facial anomalies. The statistical analyses showed that patients that were diagnosed with maxillary retrusion (p = 0.0024), maxillary vertical hyperplasia (p < 0.001), mandibular protrusion (p = 0.0031), laterogenia (p < 0.001), or craniofacial deformity (p = 0.0033) were significantly more likely to be digitally planned (Table 1). The mean number of diagnoses set per patient was 1,458 for the traditionally planned group and 2,037 for the digitally planned group.

**Table 1:** Distribution of diagnoses in the Traditional surgical planning (TSP) group and the Virtual surgical planning (VSP) group.

Diagnosis	TSP		VSP		P
	N = 638	%	N = 224	%	p < 0.05
Retrognathic maxilla		38.1		50.0	**0.0024**
Prognathic Maxilla		1.7		0.9	>0.30
CLP		4.4		2.2	0.20
Maxillary vertical hyperplasia		3.6		12.1	**<0.001**
Maxillary vertical hypoplasia		2.4		4.5	0.17
Skeletal open bite		22.3		26.8	0.20
Retrognathic mandible		29.0		29.0	>0.30
Prognathic mandible		30.7		42.0	**0.0031**
Laterogenia		12.9		31.3	**<0.001**
Hemifacial microsomia		0.2		1.3	0.10
Craniofacial anomalies		0.5		3.6	**0.0033**
Other		5.8		7.1	>0.30

TSP: traditional surgical planning; VSP: virtual surgical planning; CLP: Cleft lip and cleft palate.

**Bold indicate significant.**

Some surgeries were more frequently used in the digitally planned group, LFI (p < 0.001), maxillary segmentation (p = 0.013), BSSO (p < 0.001) and genioplasty (p = 0.0026).

Out of the 224 patients that were digitally planned, 142 (63.39%) had bimaxillary surgery (LFI + BSSO). The mean number of surgical procedures performed per patient was 1,398 for the traditionally planned group and 1,914 for the digitally planned group (Table 2).

**Table 2:** Distribution of surgical interventions in the Traditional surgical planning (TSP) group and the Virtual surgical planning (VSP) group.

Surgical interventions	TSP		VSP		P
	N = 638	%	N = 224	%	p < 0.05
LF I		47.3		72.8	**<0.001**
LF segmented		8.5		14.7	**0.013**
LF II		0.2		0.0	>0.30
LF III		0.2		0.4	>0.30
Maxillary distraction		1.1		0.9	>0.30
SARME		8.5		0.0	**<0.001**
IVRO		1.9		3.1	>0.30
EVRO		2.4		0.0	**0.022**
BSSO		61.1		85.3	**<0.001**
Segmented mandible		1.1		0.0	0.24
Mandibular distraction		0.0		0.9	0.10
Genioplasty		3.9		9.8	**0.0026**
Prosthesis		0.0		0.4	>0.30
Other		3.6		3.1	>0.30

TSP: traditional surgical planning; VSP: virtual surgical planning; LF: Le Fort; BSSO: Bilateral Sagittal Split Osteotomy; IVRO: Intraoral vertical ramus osteotomy.

**Bold indicate significant.**

For the surgical outcome analysis, only patients who underwent bimaxillary surgery without additional procedures were included. When comparing surgical time and bleeding in traditionally planned bimaxillary (LFI + BSSO) patients to digitally planned bimaxillary patients, digitally planned patients had a reduced surgical time with a mean of 23 minutes (p = 0.0053). Bleeding during surgery was lower (p = 0.054) in the digitally planned group with an average reduction of 53 mL (15.68%) per surgery, although not statistically significant. (Table 3) A separate analysis of one of the clinics with a relatively large volume of orthognathic cases was performed (25.45% of the registered bimaxillary surgeries in NROK), which showed considerably less impact on surgical time with only a 2-minute reduction with the digitally planned patients without statistical significance (p > 0.3). At the same clinic, the bleeding was reduced with 41 mL (12.9%), also without statistical significance (p > 0.3) (Table 4).

**Table 3:** Comparison of surgical time (left) and bleeding volume (right) between traditionally and digitally planned bimaxillary surgeries.

Planning	Time (minutes)				Bleeding (milliliter)			
	N	Mean	SD	Range	N	Mean	SD	Range
TSP	147	226	73	98–540	142	338	183	70–1,100
VSP	125	203	58	105–500	123	285	252	60–2,000
p < 0.05	**0.0053**				0.054			

TSP: traditional surgical planning; VSP: virtual surgical planning.

**Bold indicate significant.**

**Table 4:** Surgical time and bleeding, with traditional surgical planning (TSP) and Virtual surgical planning (VSP), during bimaxillary surgery in one high-volume center.

Planning method	N (cases)	Mean time (min)	SD	Range (min)	N (cases)	Mean bleeding (mL)	SD	Range (mL)
TSP	36	193	31	132–261	36	318	206	70–1,100
VSP	34	191	39	105–288	34	277	253	60–1,400
P		>0.30				>0.30		

TSP: Traditional Surgical Planning; VSP: Virtual Surgical Planning. P-values calculated using Fisher’s permutation test. No statistically significant differences found.

## Discussion

The aim of this study was to investigate the impact of VSP versus TSP using the Swedish NROK registry and more specifically to clarify which diagnoses most often led to the usage of VSP and also which different types of surgeries are performed when VSP has been used. Finally, an analysis was made on which clinical implications VSP has on surgical time and bleeding during surgery.

The results of this study indicate that patients where VSP was used have a higher number of dentofacial diagnoses per patient than those traditionally planned. The data also showed that the VSP patients had gone through more surgical interventions and that bimaxillary surgeries were more common in this group of patients. When surgical time and bleeding during surgery were studied in patients that underwent bimaxillary surgery, the VSP group had shorter surgical time and less bleeding during surgery compared to the TSP group, although the latter not statistically significant.

There are few studies published regarding which diagnoses or surgical treatment options correlate with the decision to use VSP. However, it is likely that the clinician would prefer to use VSP to a larger extent in more complex cases [10]. In the current study, it has been shown that patients that have been planned in a virtual environment had more diagnosed anomalies, which could indicate that the clinician considered these patients as more complex. These patients also required a larger number of surgical interventions, which may indicate greater complexity of these cases. However, this can also be interpreted as the virtual environment in itself creating a need for more surgical interventions, as the three-dimensional view gives the surgeon more information on the effect produced by different movements of bone segments.

In this study, none of the Le Fort II (LFII), SARME (Surgically-assisted rapid maxillary expansion), EVRO (Extraoral vertical ramus osteotomy), and mandibular segmental osteotomy were digitally planned. The volume of these surgeries was also relatively small in this material, wherefore it is not possible to rule out that VSP could have an impact on bleeding during surgery or the time spent in the operation room in these cases. When analyzing the volume of bimaxillary surgeries in this material, it is consistent with other similar studies [13].

To date, there is a paucity of studies published on orthognathic surgery where the effect of VSP on operative times and bleeding is evaluated. To the best of our knowledge, there has only been one study comparing VSP and TSP when it comes to perioperative bleeding, which showed less bleeding for VSP, although not statistically significant [16]. The same study also showed a statistically significant shortened operative time with VSP compared to TSP [16]. Another systematic review concluded that two out of the three included articles on the subject showed a statistically significant correlation in favor of VSP, while the third study could not support these findings [14].

As stated earlier, the data evaluated in this study indicate that VSP leads to shorter operation time and less bleeding during surgery; however, other important factors may play a role. When analyzing bimaxillary surgeries in this cohort, one must bear in mind that a large part of the bimaxillary surgeries that were planned in a virtual environment were performed at the larger clinics with more experience in orthognathic surgery, which might at least in part explain the shorter operative times and less bleeding during surgery. Although the results are inconclusive, it still points to the possibility that the effectiveness of VSP might in part be due to other factors and shows the importance of considering this in future multicenter studies. Given the small sample size within this subgroup, it is also possible that the study was underpowered to detect a true effect of VSP on surgical time and bleeding, leading to a non-significant result.

The present study is based on a registry of a nation’s population with a very high coverage rate. The registry also makes it possible to investigate a large number of patients while providing a representative group of patients with different ages, sex, and backgrounds. Although it should also be noted that a limitation with a registry study like the present one is that the clinicians that contribute to the registry are often not well calibrated, a factor which can negatively influence the quality of the data. Another important item that has to be considered is that the usage of VSP is still growing rapidly in Sweden, and different workflows are used at the different clinics. If there are clinics where VSP is used only occasionally, this may influence which cases are selected for virtual planning.

## Conclusion

This study shows that patients with the diagnoses of maxillary retrusion, maxillary vertical hyperplasia, mandibular protrusion, laterogenia, and craniofacial deformity were digitally planned to a larger extent than other orthognathic diagnoses. The findings of this study also indicate that virtual planning is being used more frequently in complex cases with a larger number of diagnosed anomalies and in need of extensive surgical interventions.
